# Beyond the Underground: Emergency Geriatric Hospital Relocation in Conflict - A Healthcare Innovation Model

**DOI:** 10.1093/geroni/igaf122.093

**Published:** 2025-12-31

**Authors:** Sharon Ost-Mor, Elena Titelman, Eduard Zlyesov, Inna Shugaev

**Affiliations:** University of Haifa, Haifa, HaZafon, Israel; Fliman Medical Center, Haifa, HaZafon, Israel; Fliman Medical Center, Haifa, HaZafon, Israel; Fliman Medical Center, Haifa, HaZafon, Israel

## Abstract

The unprecedented emergency relocation of Israel’s largest rehabilitation facility in the north, Fliman Medical Center, to an underground facility during active conflict presents a unique case study in healthcare emergency management. This research addresses a critical gap in the literature on emergency preparedness for specialized healthcare facilities during crisis situations. This qualitative single-case study examines the strategic planning and execution of relocating individuals requiring ventilator support and other complex medical care amid active conflict. Data were collected through in-depth interviews with the emergency coordinator and analysis of contemporaneous documentation. Over eleven months of security challenges, the facility successfully implemented a carefully coordinated evacuation plan, safely transferring sixty medically complex patients—along with essential medical equipment and personnel—to an underground facility at Rambam Hospital within three hours. The relocation maintained operational independence and ensured the continuity of comprehensive rehabilitation services. The successful execution of this emergency relocation protocol demonstrates the feasibility of sustaining complex medical care during crisis situations. This study offers a model for emergency preparedness in specialized healthcare facilities, providing critical insights for institutions worldwide—particularly those serving medically complex populations in conflict-prone regions. The findings present practical guidelines for the safe relocation of vulnerable patients during crises, contributing to improved healthcare resilience in emergency settings.

